# The Role of Cytoreductive Surgery in Platinum-Resistant Ovarian Cancer (PROC): A Systematic Review

**DOI:** 10.3390/cancers17020217

**Published:** 2025-01-11

**Authors:** Michail Sideris, Kshitij Jamdade, Hajar Essangri, Shruti Zalawadia, Samuel George Oxley, Kagan Selek, Saurabh Phadnis

**Affiliations:** 1 Wolfson Institute of Population Health, Cancer Research UK Barts Centre, Queen Mary University of London, Charterhouse Square Campus, Barbican, London EC1M 6BQ, UK; s.oxley@qmul.ac.uk; 2 Department of Gynaecological Oncology, Barts Health NHS Trust, Royal London Hospital, London E1 1FR, UK; hajar.essangri@nhs.net (H.E.); s.zalawadia@nhs.net (S.Z.); kagan.selek@nhs.net (K.S.); s.phadnis@nhs.net (S.P.); 3 Department of Gynaecological Oncology, Nottingham University Hospitals NHS Trust, City Hospital, Nottingham NG5 1PB, UK; kshitij.jamdade@nhs.net; 4 Department of Gynaecological Oncology, Barts Health NHS Trust, Whipps Cross Hospital, London E11 1NR, UK

**Keywords:** platinum-resistant ovarian cancer (PROC), cytoreductive surgery (CRS), progression free survival (PFS), overall survival (OS)

## Abstract

Background/Objective: Platinum-resistant ovarian cancer (PROC) has limited therapeutic options, and the role of cytoreductive surgery (CRS) in improving survival outcomes remains uncertain. We performed a systematic review to evaluate the oncological benefit of CRS on PROC patients and the associated surgical morbidity and mortality. Methods: We followed a prospective protocol according to PRISMA guidelines. We searched PubMed, Medline, and Embase till October 2024. We used a “Population Intervention Comparator Outcomes (PICO)” framework. Our population included women with epithelial PROC who underwent CRS with/without chemotherapy. Our outcomes included overall survival (OS), progression-free-survival (PFS), post-operative morbidity and mortality and Quality of Life. Results: Our search yielded 6590 citations; six studies (N = 155 patients) were included. There is limited evidence available on the role of CRS in PROC, with notable variation in reported outcomes and outcomes’ measures; therefore, we were unable to perform quantitative synthesis. CRS demonstrated survival benefits in well-selected PROC patients, particularly those with limited, isolated recurrences, low tumour burden, and good performance status. Complete resection (R0) was associated with significantly longer OS/PFS compared to those who had suboptimal surgeries (R1/R2). Conclusions: CRS seems to extend survival in carefully selected PROC patients, especially those with limited disease spread and favourable surgical profiles. Nevertheless, CRS carries substantial surgical risks, and its benefits appear contingent upon achieving R0. Further prospective trials with standardised patient selection criteria are needed to define CRS’s role in PROC. At present, CRS in PROC should be considered within a multidisciplinary approach in specialised gynaecological oncology centres, with the careful assessment of patient-specific risk factors and potential for R0 resection.

## Simple Summary

Ovarian cancer, which is resistant to platinum-based chemotherapy, has limited therapeutic options, and there is uncertainty over the role of surgery to remove all visible tumour cells (cytoreductive surgery), and its survival benefits. We performed a systematic review to evaluate the oncological benefit of this surgery in patients with platinum-resistant ovarian cancer and the associated surgical morbidity and mortality. Although evidence is limited, surgery appears to prolong survival in these patients, particularly with isolated recurrences and where there has been a full resection of all visible tumour. However, surgery is associated with substantial risks and limited benefits when all visible tumours cannot be removed. There is a need for prospective clinical trials in these patients.

## 1. Introduction

Ovarian cancer (OC) is the third most common gynaecological cancer worldwide and the most lethal [1]. This is partially attributed to patients’ late presentation and diagnosis at an advanced stage, as well as the delayed and nonspecific onset of symptoms [2]. GLOBOCAN estimates around 313,959 new cases and 207,252 deaths caused by ovarian cancer in 2020 [3]. Epidemiology of the disease is affected by socioeconomic and genetic factors; non-Hispanic white women are shown to have the largest incidence; however, the highest mortality is seen in African populations. Family history and environmental and epidemiologic factors have been associated with the disease; however, the aetiopathogenesis remains equivocal [4]. Around 15–20% of OC are hereditary-associated with moderate or high-risk cancer susceptibility genes (CSGs); common examples are the BRCA1/2 or the mismatch repair (MMR) genes in the case of Lynch Syndrome (MSH6, MLH1, MSH2 and PMS2) [5]. Upfront or interval cytoreductive surgery combined with chemotherapy is the standard of care for the management of advanced ovarian disease [6]. Complete cytoreduction, i.e., the removal of all visible disease is associated with prolonged survival and improved outcomes [7]. Neoadjuvant or adjuvant IV platinum/taxane-based chemotherapy is the standard approach, with curative, or palliative intent depending on the extent of disease on presentation [8].

Despite this aggressive approach to treatment, a significant number of patients will inevitably develop recurrent disease. Platinum-resistant OC (PROC) was historically defined as the relapse of disease within 6 months of the last cycle of chemotherapy; however, this is now used to refer to a broader group of OC which progresses within 6 months after multiple lines of chemotherapy [9]. Several treatment strategies have been investigated for PROC, including extensive secondary cytoreductive surgery with further chemotherapy. Cytoreductive surgery has long been established as a cornerstone of treatment for ovarian cancer, particularly in the setting of platinum-sensitive disease [10]. However, its efficacy and utility in cases of platinum resistance remains a subject of debate and ongoing investigation. There is consensus that the need for novel chemotherapeutic agents [11,12] is vital to improve oncological outcomes in PROC [13,14].

While studies support that CRS may offer palliative benefits, such as symptom relief and the prolongation of progression-free survival (PFS), the exact overall impact on clinical outcomes remains unknown [15]. Given this paucity of robust evidence—if not the very limited availability of it—there remains a question regarding the role of cytoreductive surgery with or without chemotherapy versus systemic treatment in terms of overall or progression-free survival. It is also important to consider the significant morbidity and mortality impact of these procedures, as well as long-term quality of life (QoL) outcomes.

We performed a systematic review to summarise published evidence on the role of CRS in epithelial PROC. This included the effect on overall survival, PFS as well as associated surgical morbidity, mortality and impact on quality of life.

## 2. Methodology

We conducted a systematic review using a prospectively designed protocol and reported our study in line with the Preferred Reporting Items for Systematic Reviews and Meta-analyses (PRISMAs).

### 2.1. Literature Search

We searched PubMed, Medline and Embase from inception to October 2024 using a pre-defined search strategy (File S1). The search was restricted to studies with English titles and conducted on human subjects. We also manually reviewed the reference lists of relevant primary studies and review articles.

### 2.2. Inclusion Criteria

**Figure 1:**
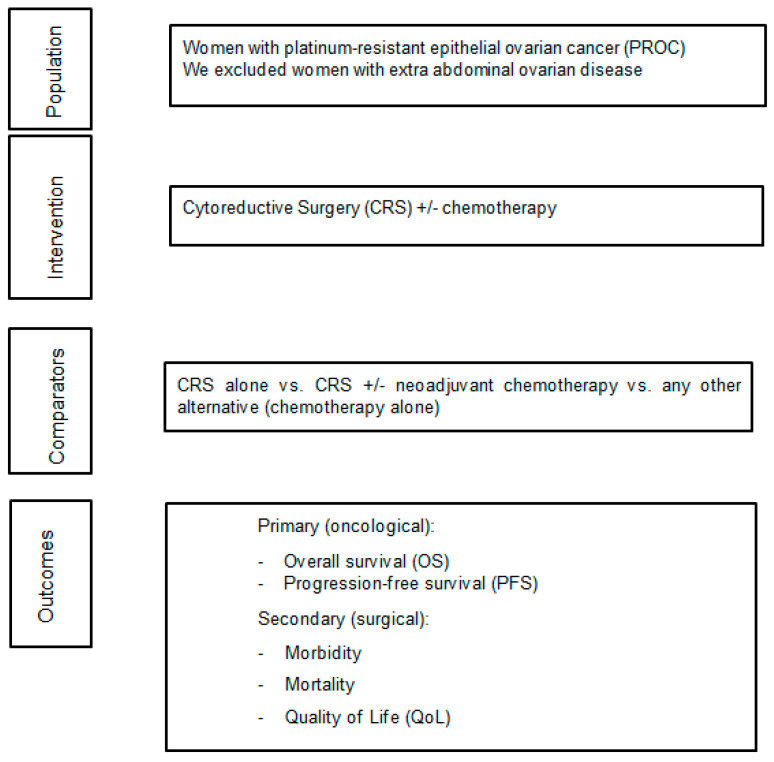
Systematic review PICO framework.

We used a “Population Intervention Comparator Outcomes (PICO)” framework to determine our inclusion criteria (Figure 1). Our population included women with PROC. We were open to report any different definition of PROC if a legitimate justification was provided. For the intervention, we mainly focused on cytoreductive surgery with or without neo/adjuvant chemotherapy. Our comparators included the administration of chemotherapy alone vs. cytoreductive surgery (with or without neo +/– adjuvant chemotherapy) vs. any other alternative treatment approach. Our primary outcomes (oncological) included overall survival (OS) and progression free survival (PFS); our secondary outcomes (surgical) included postoperative morbidity, mortality and QoL.

### 2.3. Exclusion Criteria

We excluded studies discussing women with non-epithelial ovarian cancer or women with radiological evidence of extra-abdominal metastases. Studies which did not report on at least one of our primary (oncological) outcomes were also excluded.

### 2.4. Data Selection

Titles, abstracts and full texts were screened by 3 independent reviewers (KJ/HE/KS). Any disagreements were resolved by one of the senior authors (MS/SP). Titles were handled on EndNote citation software version 18 (Clarivate).

### 2.5. Data Extraction

MS designed a Microsoft Excel extraction spreadsheet. Data were extracted by 2 pairs of independent reviewers KJ/KS and HE/SZ. Any disagreements were resolved by one of the senior authors (MS/SP). We extracted data on study design, population demographics including histology, PROC definition, types of cytoreductive surgery and chemotherapy administration, resection status (R0/R1/R2) as well as data on our reported outcomes. For qualitative synthesis, we aimed to present a table with study characteristics. Further to this, we summarised the oncological outcomes and our surgical outcomes separately using a comprehensive framework based on our PICO. Our initial agreement was to attempt quantitative synthesis only if we manage to extract data for >500 patients for each of the primary or secondary outcomes to draw meaningful conclusions. Given significant anticipated heterogeneity in reported outcomes, the sparce evidence and the limitations of the aggregate date, we decided not to proceed with a meta-analysis.

### 2.6. Quality Assessment

Two reviewers (KJ/KS) independently assessed the methodological quality of included studies using the Methodological Index for Non-Randomized Studies (MINORS) [16], with any discrepancies resolved by one of the senior authors (MS/SP). A 3-point scale graded the quality of each item, ranging from 0 (not reported), 1 (reported but inadequate), to 2 (reported and adequate). The maximum global score is 16 for noncomparative (8 items) and 24 for comparative studies (12 items). Scores ≤ 12 for noncomparative and ≤20 for comparative studies were considered to indicate high risk of bias. We also assessed the external validity of the included studies (representativeness of findings) based on whether the included population was justified with a robust definition of PROC. Studies with an unclear justification on the PROC definition were deemed high risk for external validity bias. We also assessed the external validity of the included studies (representativeness of findings) based on whether the included population was justified with a robust definition of PROC. Studies with an unclear justification on the PROC definition were deemed high risk for external validity bias.

## 3. Results

**Figure 2:**
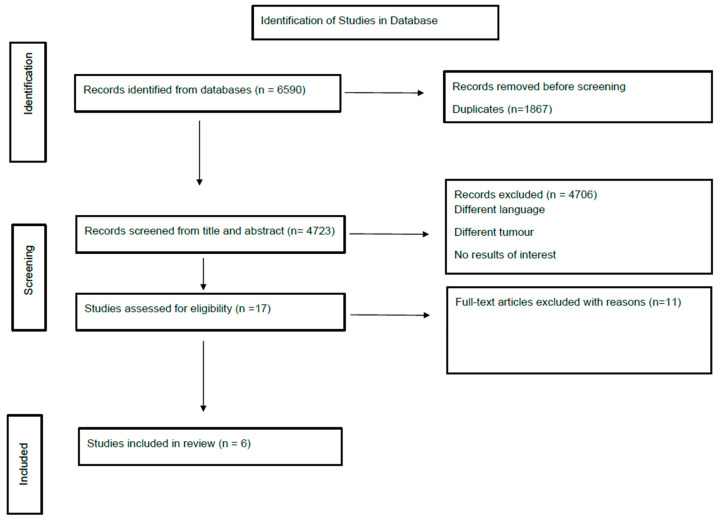
PRISMA flow diagram [17].

Our search yielded 6590 citations; six studies (N = 155 patients) were included in our narrative synthesis. Figure 2 illustrates the article selection process. There is limited evidence available on the role of CRS in PROC; we were unable to perform quantitative synthesis.

### 3.1. Study Characteristics

All included studies were retrospective in nature, comprising three observational studies and three comparative studies with control arms, containing a total of 155 patients. Cytoreductive surgery (CRS) efficacy was compared to chemotherapy in 65 patients across the six studies. Detailed study characteristics are demonstrated in Table S1.

### 3.2. Definition of Platinum-Resistant OC (PROC)

Definitions of PROC varied across studies. Zou et al. [18] defined platinum resistance as progression within six months of the last chemotherapy dose and considered progression within four weeks as “platinum-refractory”. Petrillo M. et al. [19], Tunninetti V. et al. [20] and Musella A. et al. [21] agreed on defining platinum resistance as a platinum-free interval of six months or less. Zhao [22] specified platinum-resistant recurrent epithelial OC as progression within six months or failure to control the disease post-chemotherapy. On the other hand, Le et al. [23] used a biochemical profile of less than 50% CA-125 reduction after three cycles to define PROC.

### 3.3. Oncological Outcomes

Three out of the six studies reported oncological outcomes following SCRS in comparison to a control arm, including PFS, post-relapse survival (PRS), and OS. Petrillo et al. [19] compared patients undergoing SCRS + non-platinum-based chemotherapy vs. non-platinum-based chemotherapy alone, and they found a longer PRS in the surgical arm of 32 months vs. 8 months, respectively (*p* = 0.002). Zou et al. [18] reported a longer PFS in the surgery + chemotherapy arm compared to the chemotherapy arm (10.6 months vs. 5.1 months, *p* = 0.0035) with an overall PRS of 32.6 months vs. 16.3 months, respectively (*p* = 0.047). Musella et al. [21], on the other hand, compared outcomes post-SCRS to a historical cohort with similar characteristics who were not eligible for surgery, demonstrating a higher median OS of 67 months vs. 24 months (*p* = 0.034) and a 5-year OS of 57 vs. 23.5 months (*p* = 0.035) for the surgery and chemotherapy groups, respectively (Table S2).

Three observational studies reported oncological outcomes. Le T. et al. [23] reported 17 cases of PROC; all underwent CRS, followed by three or four additional cycles of platinum-taxane chemotherapy. Reduction in residual disease was associated with an improved response to primary combined treatment (*p* = 0.007), along with enhanced platinum sensitivity at the first clinical recurrence (*p* = 0.02). An Italian cohort (Tunninetti V. et al. [20]) described 50 patients with PROC (20 primary and 30 secondary resistance); of these, 18 patients had CRS without pre-operative chemotherapy, while 32 received chemotherapy first. Complete cytoreduction (R0) was achieved in 54% of cases and was associated with a higher median OS (32.9 months vs. 4.8 months for suboptimal surgery; *p<* 0.001). Zhao et al. [22] described R0 resection in 25 patients compared to 13 with residual disease. There was higher PFS (12 vs. 8 months; *p* = 0.001) and OS (39 vs. 15 months; *p* = 0.021) in the R0 + chemotherapy group compared to the incomplete resection group (R1/2) (Table S3).

### 3.4. Surgical Morbidity Post-CRS for PROC

Included studies reported on complications such as intraoperative visceral injuries, wound dehiscence, and postoperative infection requiring intensive care admission. Zou R. et al. [18] compared SCRS vs. chemotherapy arms, and mortality was reported in 13/21 patients in the surgical group versus 23/31 in the chemotherapy group, over a median follow-up of 70.6 months. Tuninetti V. et al. [20] described a surgical morbidity rate of 38%, with complications such as pneumothorax (10%), acute respiratory failure (4%), lymphocele (4%), and a 30-day postoperative mortality of 8%. Table S4 summarises the morbidity and mortality associated with SCRS in PROC.

### 3.5. Quality Assessment

File S2 summarised the MINORS checklist results for the included studies. All studies were deemed high risk for bias due to the retrospective data of data collection, the lack of prospective sample sizes and over 5% loss to follow-up.

## 4. Discussion

### 4.1. Summary of Results

Our systematic review included six retrospective studies involving 155 patients undergoing CRS for PROC [18,19,20,21,22,23]. There is variation in PROC definitions with most authors leaning towards a disease-free interval of no more than 6 months and others using a biochemical profile of <50% Ca125 reduction after 3 cycles of neoadjuvant chemotherapy (NACT). The included studies collectively support that CRS may confer a survival advantage, particularly when complete resection (R0) is achievable. While most studies indicate improved survival with CRS in well-selected PROC cases, the wider role of CRS in broader PROC populations remains uncertain. Some evidence suggests that the survival benefit is largely confined to those without peritoneal carcinomatosis, those with a limited number of lesions and those with a good fitness status, indicating that CRS might be most effective when used in highly controlled scenarios. All studies were retrospective, with a high risk of bias. We did not find any data on QoL following cytoreductive surgery in the context of PROC.

### 4.2. Interpretation

#### 4.2.1. Does CRS Improve Survival in PROC?

The survival benefit of CRS in PROC has been previously debated, with mixed results on its impact on survival outcomes [18,19,20,21,22,23]. All our included studies showed that CRS combined with chemotherapy extended PFS and OS, on the condition that complete resection (R0) is achieved. There is a consensus from all six studies that, in select cases with a small volume or isolated sites of disease, CRS as the primary or adjunct treatment modality (with chemotherapy) offers significant survival benefits.

Le T. et al. [23] observed that patients who showed limited reduction (<50%) in CA-125 after chemotherapy benefited from radical debulking surgery regardless of their response to NACT followed by 3–4 additional cycles of platinum-taxane chemotherapy. The study observed a significant reduction in residual disease and improved response to primary combined treatment, along with enhanced platinum sensitivity at the first clinical recurrence. This potentially supports a more evolutionary approach of offering CRS in early platinum resistance to improve the effectiveness of subsequent platinum-based chemotherapy, potentially extending progression-free and overall survival.

However, across all studies, the survival advantage appears limited to patients with specific clinical and oncological characteristics. CRS is less beneficial for those with extensive disease spread or poor performance status, as the substantial surgical risks may outweigh potential gains. Thus, while CRS may extend survival in PROC, its use is best restricted to patients most likely to achieve R0 resection and to experience a significant survival benefit. A recent meta-analysis showed a clear survival benefit with acceptable morbidity in the setting of secondary, tertiary or quaternary CRS, supporting the pivotal role of CRS in treating recurrent OC.

#### 4.2.2. CRS in PROC—Who Is Suitable?

The feasibility of CRS in PROC is closely tied to patient selection and specific disease factors. Patients with isolated nodal recurrence or single-site lesions are most likely to achieve complete resection, which is crucial for meaningful survival benefit [19,22]. Achieving R0 resection has been highlighted as a pivotal factor in CRS’s success, as patients with incomplete resection generally experience limited survival benefit. Factors which enable this include isolated nodal recurrence or single anatomic lesions, the absence of peritoneal carcinomatosis, and good performance status (ECOG 0-1) [19,21,22].

Some studies explored the role of minimally invasive surgery (MIS) approaches, including laparoscopy [18,22]. In the era of robotic surgery, MIS has the potential to minimise potential morbidity if relevant expertise is available. It may also have a role in patient selection, including determining the feasibility of CRS and hence preventing unnecessary laparotomy and associated perioperative morbidity. However, given the surgical complexity of these patients, anatomic location and extent of disease, MIS approaches may be too optimistic at present [24].

#### 4.2.3. When and How Is CRS a Safe Option in Practice?

Achievability relates to the practical considerations of conducting CRS in patients with PROC safely. This can be constrained by the elevated risk of surgical-related morbidity and mortality, especially in heavily pre-treated PROC patients. The studies conducted by Zhao et al. [22] and Tunninetti et al. [20] documented notable risks, such as VTE, intraoperative vessel injury, and wound dehiscence. Zhao et al. [22] reported high morbidity in cases where complete resection was challenging, leading to prolonged hospital stays and high postoperative morbidity. Tunninetti et al. [20] further highlighted that 38% of patients experienced morbidity, with some requiring intensive care for issues such as pneumothorax and acute respiratory failure.

These findings do not discourage us from offering surgery in these patients as they may be similar to what we are used to in CRS for AOC [25,26]. However, they do imply that, while CRS may be achievable, it still necessitates strict patient selection criteria, a skilled surgical team, and access to multidisciplinary comprehensive postoperative care to mitigate risks. It also requires support from a multidisciplinary cancer network who would carefully select those patients that have exhausted all other safe alternatives. Understanding and appreciating the complexity of such procedures, CRS in PROC should likely be reserved for patients with low surgical risk profiles who are anticipated to benefit most from potential survival gains. Given these complexities, CRS for PROC is most suitable in supra-regional specialised GO centres with advanced perioperative care facilities to manage these high-risk patients.

#### 4.2.4. Strengths/Limitations

We used a systematic review protocol to identify all published evidence on PROC. We appraised the evidence using a validated risk-of-bias tool and evaluated the representativeness of the findings of each cohort study across a global setting. Nevertheless, the findings of this review are limited by the limited data. Our included studies were mostly retrospective in design, which can introduce selection bias and inconsistencies in treatment protocols. This is reflected by all studies being deemed at high risk for bias. Additionally, the small sample sizes and heterogeneous definitions of platinum resistance across studies complicate direct comparison and limit the generalizability of these results. Given the complexity of the topic, these data can only be used as a suggestion for future research and stimulation for discussion. There is a paucity of evidence on the role of variations in reported morbidity and mortality across different ethnic backgrounds.

## 5. Conclusions

In conclusion, this review highlights that CRS may offer a survival benefit for selected patients with platinum-resistant ovarian cancer, particularly those with limited disease and favourable performance status. While promising, the efficacy of CRS in PROC remains conditional on achieving complete resection and mitigating the considerable surgical risks. The complications associated with CRS underscore the need for stringent patient selection and careful peri-operative planning. Future research should focus on prospective trials to better define the role of CRS and identify biomarkers predictive of benefits in PROC. For now, CRS may be considered in specialised centres with expertise in ovarian cancer surgery, and only after thorough patient evaluation and counselling about potential risks and benefits. As such, CRS is not yet a definitive standard in PROC but could be a valuable tool within a multidisciplinary approach for carefully chosen cases. Future research should focus on validating patient selection criteria, prospectively determining the impact of chemotherapy prior to CRS on oncological outcomes and developing less invasive surgical techniques like robotic or laparoscopic surgery to expand CRS’s feasibility. The paucity of robust evidence necessitates larger prospective trials to establish robust data in order to standardise protocols for CRS in PROC and guide clinical decision making more effectively. Further to this, considering adding QoL outcomes, as well as including a diverse ethnic population in the trial design would be of paramount importance in generating valuable evidence on the topic.

## Data Availability

Dataset available upon request from the authors.

## Supplementary Materials

The following supporting information can be downloaded at https://www.mdpi.com/article/10.3390/cancers17020217/s1, Table S1: Study characteristics; Table S2: Synthesis of oncological outcomes in studies comparing SCRS to a control arm; Table S3: Oncological outcomes in studies with no control arm; Table S4: Morbidity and mortality outcomes; File S1: Search Strategy; File S2: MINORS Quality Assessment checklist. References [18,19,20,21,22,23] are cited in the Supplementary Materials.
