# Supplementary material for: The Role of Cytoreductive Surgery in Platinum-Resistant Ovarian Cancer (PROC): A Systematic Review
Source: Cancers (Basel). 2025 Jan 11;17(2):217. doi: 10.3390/cancers17020217 (PMC11764277; doi:10.3390/cancers17020217)
Supplement: Supplementary file 1 [file cancers-17-00217-s001.zip › cancers-3334493-supplementary.pdf]

Table S1: Study characteristics.

First author	Year of publication (Country)	Study design	Sample size (n)	PROC definition	Scope
Zou R. et al [18]	2023 China	Retrospective cohort	21 SCRS vs 31 Chemo	Disease progression within 6 months from the last dose of platinum chemotherapy as "platinum resistance" Disease progression within 4 weeks from the last dose of platinum chemotherapy as "platinum refractory".	To evaluate the efficacy of SCRS versus Chemo for the treatment of limited regional PROC
Le T et al [23]	2009 Canada	Retrospective	17 SCRS	Decrease in serum CA-125 of less than 50% after three cycles of chemotherapy from the time of initial diagnosis.	To assess the impact of interval surgical debulking in women with OC who showed 'biochemical' resistance to neoadjuvant platinum-based Chemo.
Petrillo M et al [19]	2014 Italy	Retrospective	11 (SCRS + Non-P Chemo) Vs 16 (Chemo alone)	Platinum free interval (PFI) was defined as the time elapsed between the end of primary platinum-based Chemo and first recurrence, and all women showing a PFI ≤6 months were classified as platinum resistant.	To assess the impact of SCRS on survival outcome in a retrospective series of isolated* PROC<br><br>*A single nodule, in a single anatomic site
Musella A et al [21]	2015	Retrospective	18 (SCRS) Vs 18 (Chemo alone)	Disease-free interval less than 6 months.	To assess the impact of SCRS in women eligible for surgery on overall survival, in comparison to a historical series not eligible for surgery

Tuninetti V et al [20]	2020 Italy	Retrospective Observational	50 (SCRS)	Platinum free interval <6 months before recurrence	To assess outcomes of SCRS in PROC patients.
Zhau LQ et al [22]	2022 China	Retrospective Observational	38 (SCRS)	Failure to achieve disease control condition after platinum-based chemotherapy or recurrence within 6 months after discontinuation of platinum-based chemotherapy.	To evaluate the feasibility of SCRS for treating PROC

SCRS: secondary cytoreductive surgery, PROC: platinum resistant ovarian cancer, OC : ovarian cancer, Non-P chemotherapy : non platinum based chemotherapy

Table S2: Synthesis of oncological outcomes in studies comparing SCRS to a control arm.

First author, year, Country	Cohort	Surgery Arm	Control Arm	Outcomes	Results	Interpretation
Petrillo M, 2014, Italy [19]	1995-2011	11 (SCRS + Non-P Chemo)	16 (Non-P Chemo Alone)	PRS	PRS of 32 *vs* 8 months (***p***=0.002)	SCRS prolongs PRS compared to chemotherapy alone in isolated PROC.
Musella A, 2015, Italy [21]	1995 - 2013	18 (SCRS) (2005 to 2013)	18 (Chemo in historical cohort)	Median OS<br>5-year OS	OS of 67 *vs.* 24 months (***p***=0.034)<br><br>5-year OS of 57 *vs.* 23.5 months (*p*=0.035)	After careful selection, in this setting, surgery could represent a useful adjunct to chemotherapy, in highly selected centres.
Zou R 2023 China, [18]	2015-2022	21 (SCRS + Chemo)	31 (Chemo)	Median PFS<br>PRS	PFS of 10.6 *vs.* 5.1 months (***p***=0.0035)<br><br>Overall PRS 32.6 *vs.* 16.3 months (***p***=0.047)	When well-selected PROC patients with limited regional recurrence achieved R0, their outcomes were superior to those of patients who received only chemotherapy with an acceptable morbidity rate.

SCRS: secondary cytoreductive surgery, PROC : platinum resistant ovarian cancer, OC : ovarian cancer, Non-P chemo : non platinum based chemotherapy, SCRS : secondary cytoreductive surgery, Non-P Chemo : non platinum chemotherapy, PRS : Post relapse survival, OS : overall survival, PFS : progression free survival

Table S3: Oncological outcomes in studies with no control arm.

First author, year, Country	Cohort	Surgery Arm	Sub-Group (comparator)	Outcomes	Results	Interpretations
Le T, 2009 Canada [23]	1997 - 2005	17	Residual disease <1cm; 1-2 cm; >2cm	CA 125 trend Median PFI	Amount of residual disease was associated with clinical response to selected treatment approach (p=0.007) improved platinum sensitivity status at the time of first clinical recurrence (P = 0.02)	Optimal debulking has potential survival benefits in PROC.
Tuninetti V 2020 Italy [20]	2010 – 2018	50	27 (CC 0) *vs*. 21 (CC >0)<br><br>18 (SCRS) *vs*. 32 (NACT + SCRS)	median OS	Median OS of 32.9 *vs* 4.8 months HR 4.21 (p<0.001)	R0 resection of PROC is associated with longer survival.
Zhao LQ 2022, China [22]	2012 - 2018	38		Median PFS Median OS Median CFI	PFS (12 vs. 8 months; *p*=0.001) and OS (39 vs. 15 months; *p*=0.021) in the R0 + chemotherapy group compared to the incomplete resection group (R1/2)	R0 resection and postoperative chemotherapy could significantly prolong PFS and OS, while R0 resection significantly prolonged the CFI.

PFS – Progression Free Survival, OS – Overall Survival, CFI - Chemotherapy-free interval, CC-Completeness of cytoreduction, NACT – Neoadjuvant Chemotherapy, SCRS – Secondary Cytoreduction Surgery, HR, Hazard ratio

Table S4: Morbidity and mortality outcomes

First author, year, Country	N	Surgical morbidity	Surgical related mortality (30-day period reported deaths)
Petrillo M, 2014 Italy [19]	11 (CRS + Non-P Chemo)	-Asymptomatic lymphocele<br>-Wound dehiscence	No surgical related mortality reported.
Musella A, 2015 Italy [21]	18 (CRS)	-Intra-op internal iliac vein injury (1)<br>-Pulmonary embolism (1)<br>-Haemorrhage (1)<br>-Wound dehiscence (2)	No surgical-related mortality reported.
Zou R 2023 China [18]	21 (CRS + Chemo)	-Post-op infection (2)<br>- Anaemia (2)<br>-Hydroureter (1)<br>-Postoperative ileus (1)<br>- Acute severe pneumonia (1)<br>-Grade 3 or more = 3	No surgical-related mortality reported.
Le T, 2009, Canada [23]	17 (Interval CRS)	No surgical related morbidity was reported.	No surgical-related mortality reported.
Tuninetti V 2020 Italy [20]	50	-Pneumothorax (5)<br>- Acute respiratory failure (2)<br>- Lymphocele (2)<br>- Parenteral nutrition (2)<br>-Renal insufficiency and electrolyte imbalance (2)<br>- Infection/sepsis (2)<br>- Pleural effusion (1)<br>- Gastric laceration (1)<br>- Subocclusion (1)<br>-Pyelostomy (1)<br>* Rate of Morbidity 38% (19/50)	30-Day Mortality = 4 (8%)
Zhao LQ 2022 China [22]	38	Rectovaginal fistula (n = 1), Intestinal and urinary fistulas (n = 1), and Renal failure-associated death (n = 1)<br>1 – Post Op Death<br>2 – Intestinal Obstruction<br>1 – Intestinal and Ureteral Fistula<br>2- Effusion of Spleen fossa and Pelvic collection (Abscess)<br>1 – Renal dysfunction and electrolyte imbalance.	N= 0* (Surgery related deaths)<br><br>*Apart from renal failure associated death, other post-op complications were successfully managed. It is not clear if the renal failure was a result of CRS.

Chemo – Chemotherapy, CRS – Cytoreduction Surgery, Non-P Chemo: Non platinum chemotherapy, Mo - Months, FU - Follow up

File S1: Search Strategy

1.PubMed
1-Ovarian Cancer [MeSH Terms]
2-Ovarian Neoplasm [MeSH Terms]
3- Epithelial Ovarian Cancer [MeSH Terms]
4-1 or 2 or 3
5-Cytoreductive Surgery [MeSH Terms]
6-Primary Cytoreductive Surgery [MeSH Terms]
7-Secondary Cytoreductive Surgery [MeSH Terms]
8-4 or 5 or 6
9-Survival Ovarian Cancer [MeSH Terms]
10-Disease Free Progression Ovarian Cancer [MeSH Terms]
11-Progression Free Survival Ovarian Cancer [MeSH Terms]
12-7 or 8 or 9
13-Platinum Resistance [MeSH Terms]
14-Platinum-Resistant Ovarian Cancer [MeSH Terms]
15-Platinum Resistant Ovarian Cancer [MeSH Terms]
16-Platinum-Resistant Ovarian Neoplasm [MeSH Terms]
17-Platinum-Resistant Recurrent Ovarian [MeSH Terms]
18-Platinum-Resistant Recurrent Ovarian Cancer [MeSH Terms]
19-13 or 14 or 15 or 16 or 17 or 18
20- 4 and 8 and 12 and 19
21-Limit 20 to (English Language and Humans)

2.Ovid MEDLINE
1- exp Ovarian Cancer/
2- exp Ovarian Neoplasm/
3- exp Epithelial Ovarian Cancer/
4-1 or 2 or 3
5- exp Cytoreductive Surgery/
6-exp Primary Cytoreductive Surgery/
7-exp Secondary Cytoreductive Surgery/
8-4 or 5 or 6
9-exp Survival Ovarian Cancer/
10-exp Disease Free Progression Ovarian Cancer/
11-exp Progression Free Survival Ovarian Cancer/
12-7 or 8 or 9
13-exp Platinum Resistance/
14-exp Platinum-Resistant Ovarian Cancer/
15-exp Platinum Resistant Ovarian Cancer/
16-exp Platinum-Resistant Ovarian Neoplasm/
17-exp Platinum-Resistant Recurrent Ovarian/
18-exp Platinum-Resistant Recurrent Ovarian Cancer/
19-13 or 14 or 15 or 16 or 17 or 18
20- 4 and 8 and 12 and 19
21-Limit 20 to (English Language and Humans)

3.Embase Classic + Embase
1- exp Ovarian Cancer/

2- exp Ovarian Neoplasm/
3- exp Epithelial Ovarian Cancer/
4-1 or 2 or 3
5- exp Cytoreductive Surgery/
6-exp Primary Cytoreductive Surgery/
7-exp Secondary Cytoreductive Surgery/
8-4 or 5 or 6
9-exp Survival Ovarian Cancer/
10-exp Disease Free Progression Ovarian Cancer/
11-exp Progression Free Survival Ovarian Cancer/
12-7 or 8 or 9
13-exp Platinum Resistance/
14-exp Platinum-Resistant Ovarian Cancer/
15-exp Platinum Resistant Ovarian Cancer/
16-exp Platinum-Resistant Ovarian Neoplasm/
17-exp Platinum-Resistant Recurrent Ovarian/
18-exp Platinum-Resistant Recurrent Ovarian Cancer/
19-13 or 14 or 15 or 16 or 17 or 18
20- 4 and 8 and 12 and 19
21-Limit 20 to (English Language and Humans)

Search algorithm: ((((((((ovarian cancer) OR (ovarian neoplasm)) OR (epithelial ovarian cancer)) OR (cytoreductive surgery)) OR (primary cytoreductive surgery)) OR (secondary cytoreductive surgery)) OR (survival ovarian cancer)) OR (disease free progression ovarian cancer)) OR (progression free survival ovarian cancer)) AND ((((((platinum-resistant ovarian cancer) OR (platinum resistance)) OR (platinum-resistant recurrent ovarian)) OR (platinum-resistant recurrent ovarian cancer)) OR (platinum resistant ovarian cancer)) OR (platinum resistant ovarian neoplasm)

File S2: MINORS Quality Assessment checklist

MINORS Quality Assessment Table

	A Clearly Stated Aim	Inclusion of Consecutive Patients	Prospective Collection of Data	Endpoints appropriate to the aim of the study	Unbiased Assessment of the Study Endpoint	Follow Up Point Appropriate to the Aim of the Study	Loss to Follow Up Less than 5%	Prospective Calculation of the Study Size	An Adequate Control Group	Contemporary Groups	Baseline Equivalence of Groups	Adequate Statistical Analysis	Total
**Le Tien 2009**	2	2	0	2	2	2	0	0	N/A	N/A	N/A	N/A	10/16
**Petrillo M 2014**	2	2	0	2	2	2	0	0	2	2	1	2	17/24
**Musella A 2015**	2	2	0	2	2	2	0	0	2	2	2	2	18/24
**Valentina Tuninetti 2020**	2	1	0	2	2	2	0	0	N/A	N/A	N/A	N/A	9/16
**Zhao LQ 2022**	2	2	0	2	2	2	0	0	NA	NA	NA	NA	10/16
**Zou Ruoyao 2023**	2	2	0	2	2	2	0	0	2	2	1	2	17/24

The items are scored 0 (Not reported), 1 (Reported but not adequate), 2 (Reported and adequate). The global ideal score being 16 for non-comparative studies and 24 for comparative studies.
